# Supplementary material for: Artesunate treats obesity in male mice and non-human primates through GDF15/GFRAL signalling axis
Source: Nat Commun. 2024 Feb 3;15:1034. doi: 10.1038/s41467-024-45452-3 (PMC10838268; doi:10.1038/s41467-024-45452-3)
Supplement: Supplementary file 1 — Supplementary Information [file 41467_2024_45452_MOESM1_ESM.pdf]

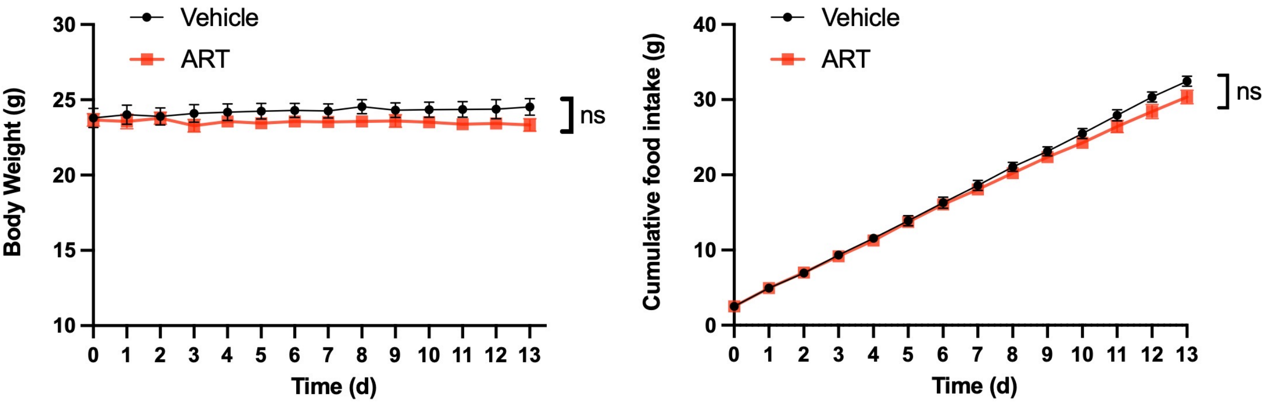

Figure S1 Artesunate (ART) treatment does not alter body weight in lean mice. Changes in body weight in wildtype mice fed chow diet upon daily intraperitoneal administrations of vehicle or ART (20 mg/kg) for 13 days (n=5 for all treatment groups). Data are presented as mean ± SEM.

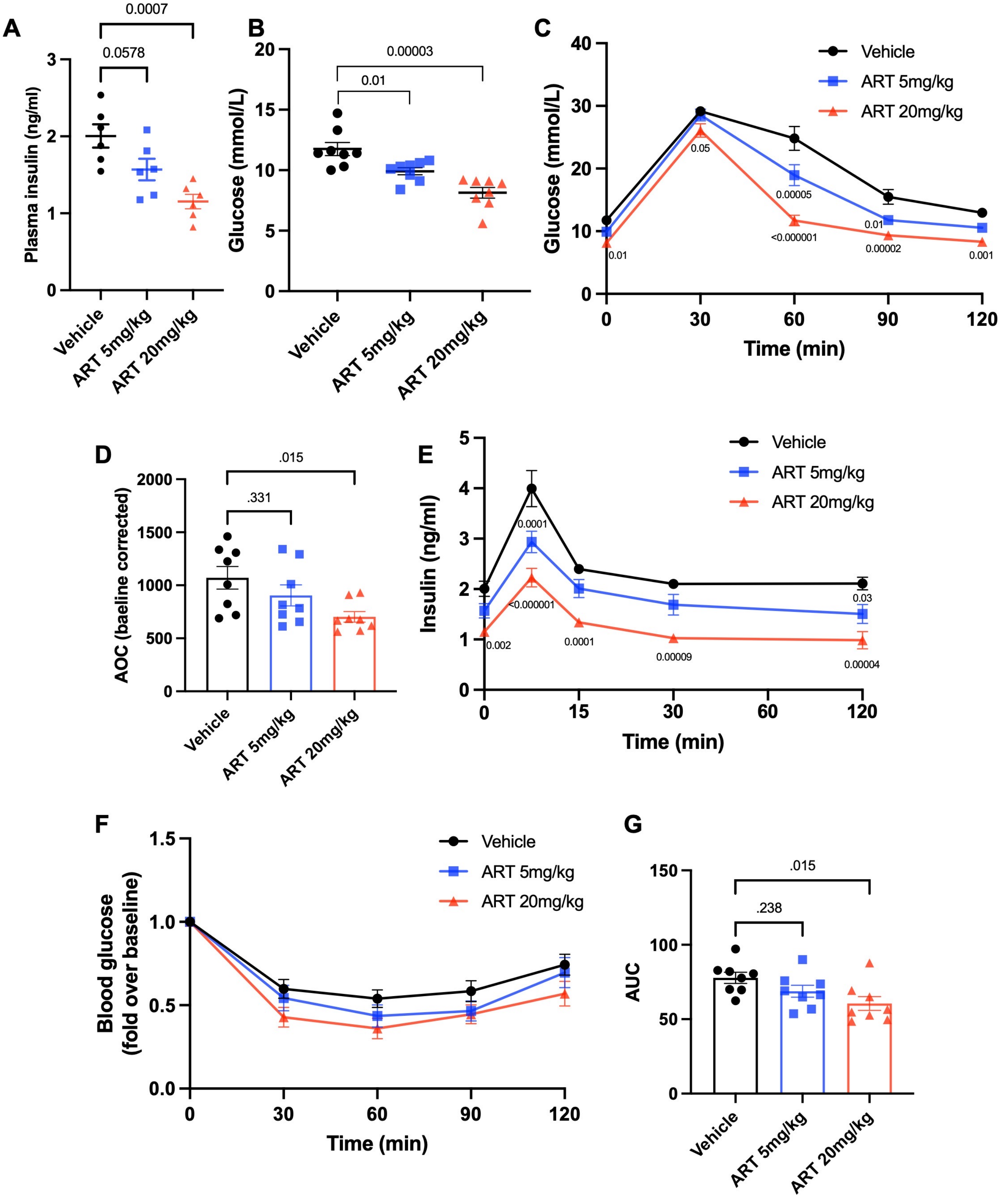

Figure S2 Artesunate (ART) treatment improves insulin sensitivity in mice with diet-induced obesity. Wild-type mice fed a high-fat diet (HFD) were treated with ART (5mg/kg and 20mg/kg) for 60 days. **(A-G)** Fasting insulin levels (n=6 for all treatment groups) **(A)**, fasting blood glucose **(B)**, plasma glucose levels during glucose tolerance test at the end of the high-fat diet challenge **(C)**, Area under curve (AUC) of plasma glucose in glucose tolerance test **(D)**, insulin levels in glucose tolerance test **(E),** plasma glucose levels during insulin tolerance test at the end of the HFD challenge **(F)**, AUC of plasma glucose in insulin tolerance test **(G)** (n=8 for all treatment groups). Data are presented as mean ± SEM. One-way ANOVA (A-B, D & G); two-way ANOVA (C, E & F).

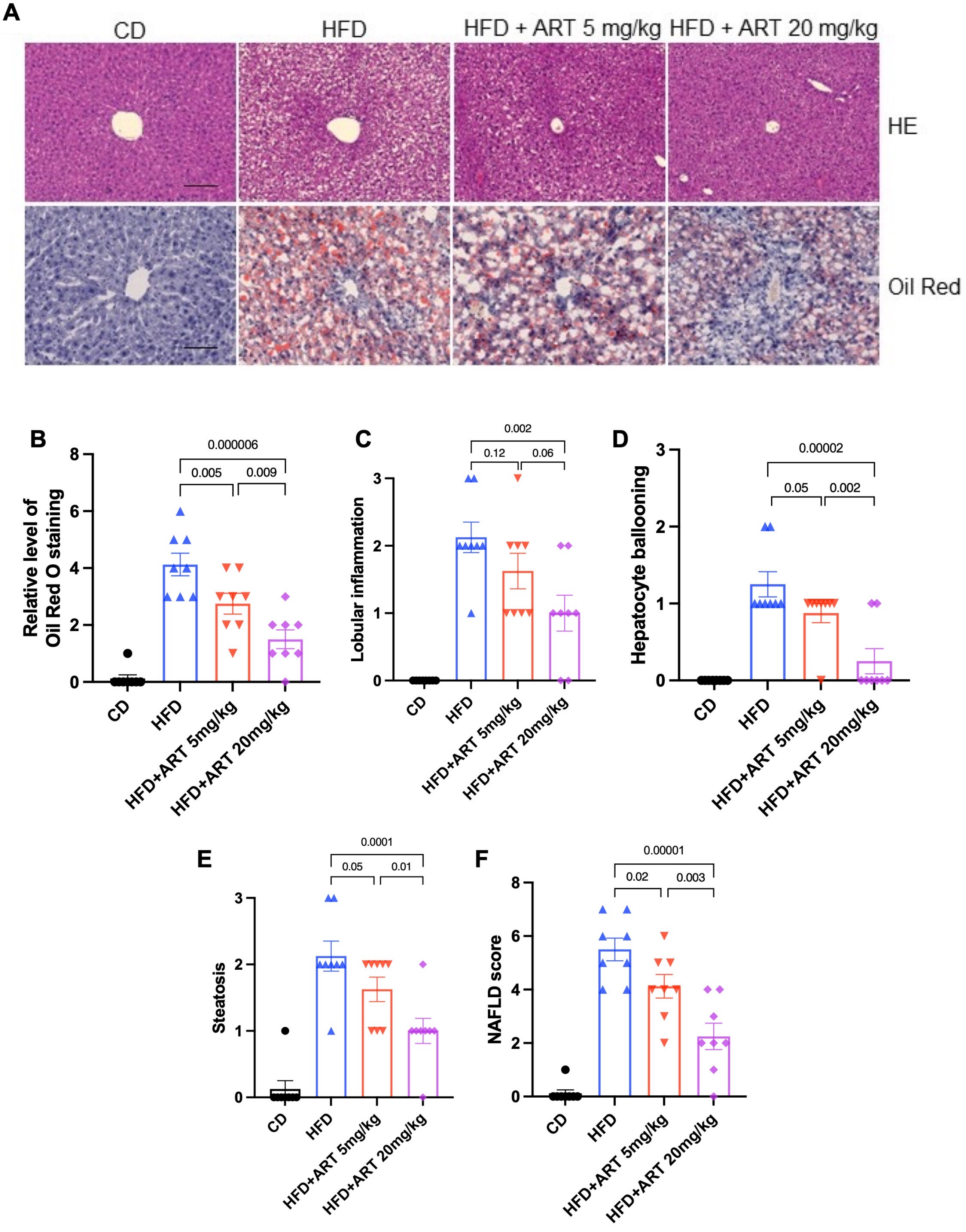

Figure S3 Chronic artesunate (ART) treatment alleviates hepatic steatosis in mice with diet-induced obesity. **(A)** Representative images showing H&E and Oil Red stained liver sections from ART-treated mice and vehicle control at the end of the high-fat diet (HFD) challenge (n = 8 per group), Scale bar 100µm. **(B)** Oil red o staining indicated reduced intracellular lipid accumulation in ART-treated mice. **(C-E)** The liver of ART-treated mice exhibited a marked reduction in lobular inflammation **(C)**, hepatocyte degeneration **(D),** and steatosis **(E). (F)** Nonalcoholic fatty liver disease (NAFLD) activity score which summarizes liver steatosis, hepatocyte ballooning degeneration, and lobular inflammation was significantly reduced in ART-treated mice. (n=8 for all treatment groups), Data are reported as average ± SEM. One-way ANOVA.

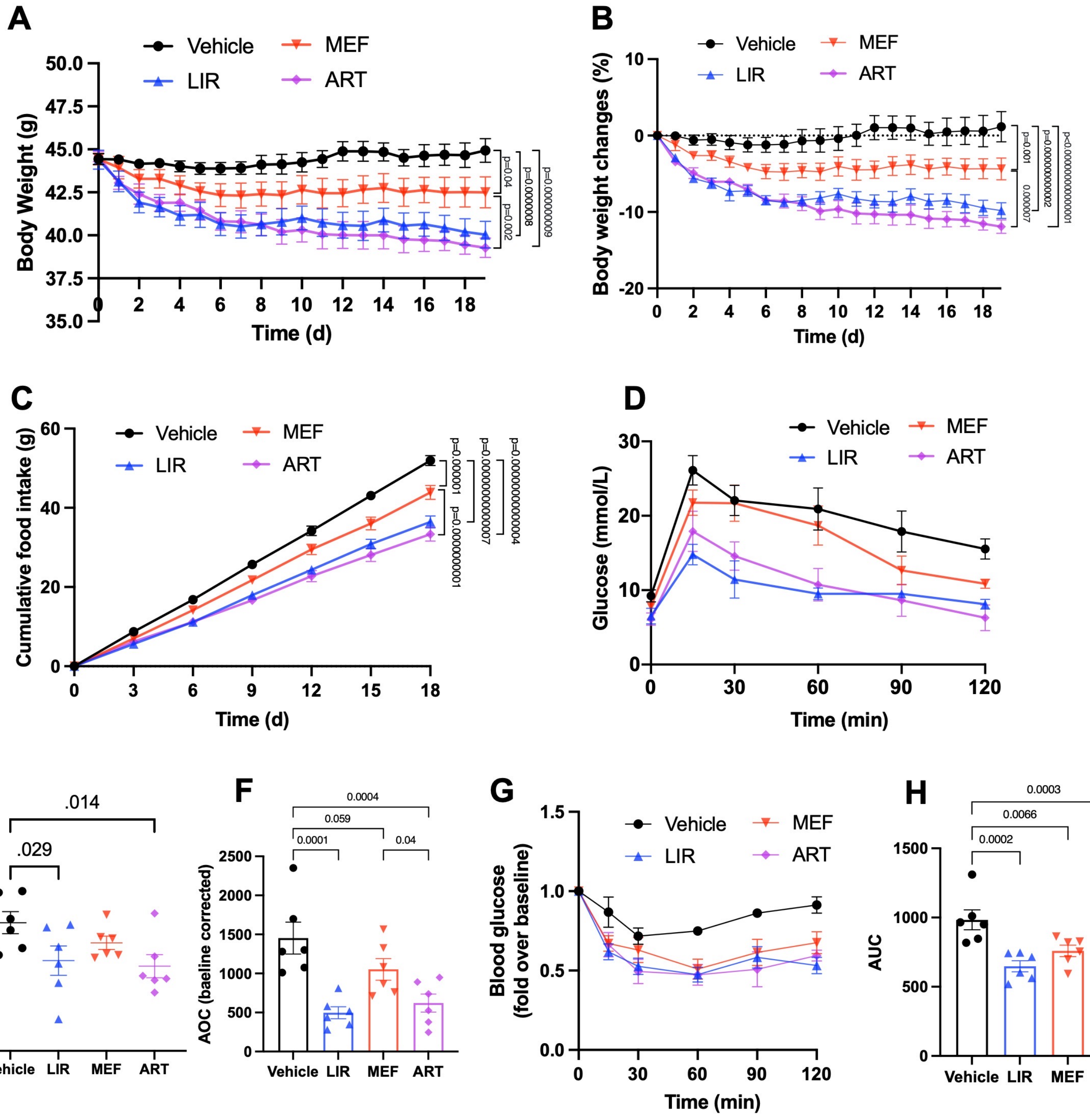

Figure S4. Artesunate (ART) is an improved anti-obesity drug in compared to metformin (MEF) and liraglutide (LIR). High fat diet (HFD) induced obese mice were treated with MEF (200mg/kg), LIR (100 ug/kg) or ART (20mg/kg) every alternate day till the end of the experiment. **(A-C)** Changes in body weight **(A)**, percentage change in body weight **(B),** and cumulative food intake of vehicle or drug (MEF/LIR/ART) treated mice **(C). (D-G)** glucose tolerance test (GTT) **(D)**, fasting plasma glucose **(E)** area under curve (AUC) during the intraperitoneal GTT **(F),** insulin tolerance test (ITT) **(G),** AUC based on the glucose level at each time point after exogenous insulin injection **(H)**. Data are reported as average ± s.e.m. Two-way ANOVA (A-D & G); One-way ANOVA (E-F, & H);

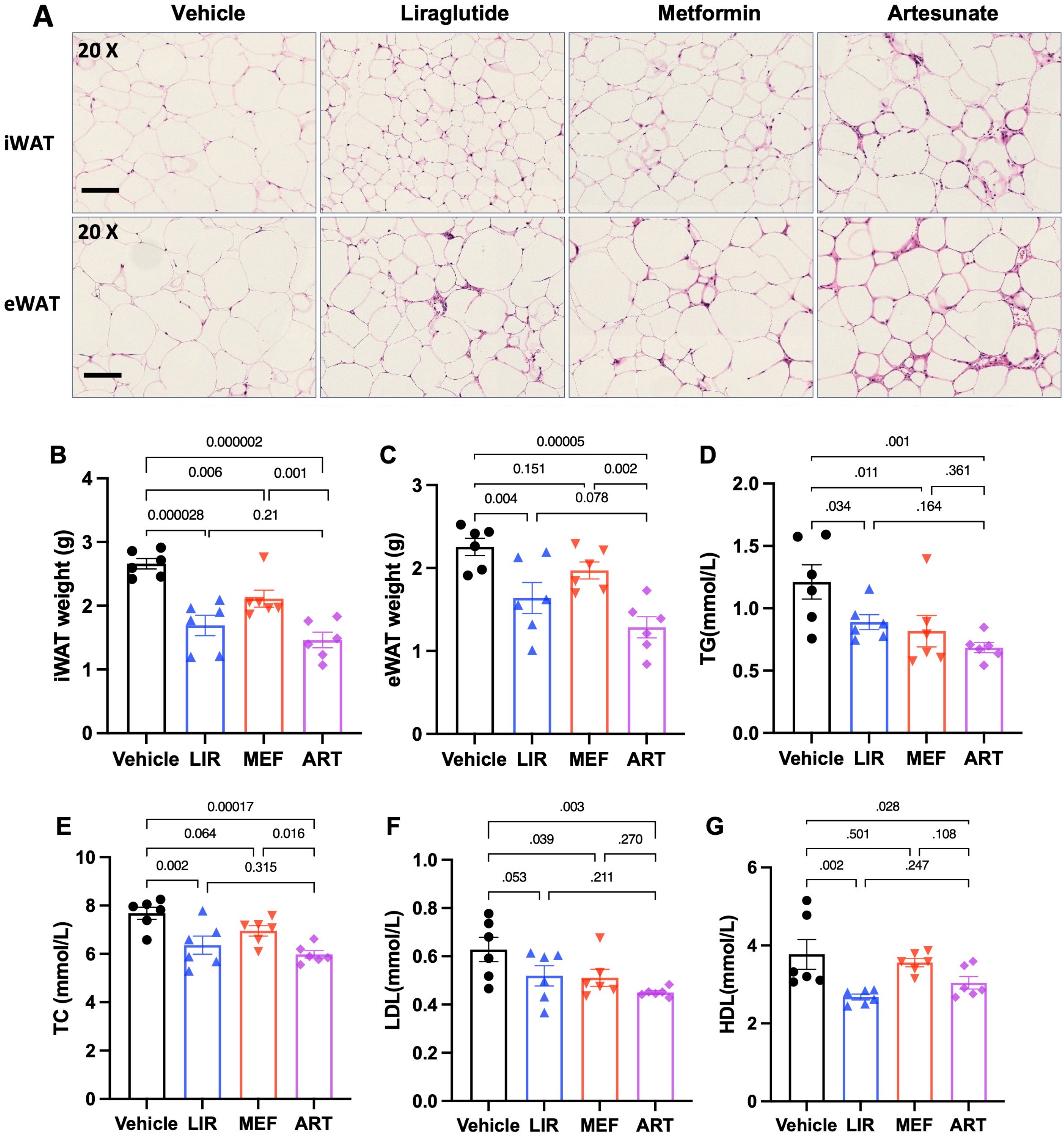

Figure S5 Artesunate (ART) improves lipid homeostasis as compared to metformin (MEF) and liraglutide (LIR). High-fat diet (HFD) induced obese mice were treated with MEF (200mg/kg), LIR (100 ug/kg) or ART (20mg/kg) every alternate day till the end of the experiment. **(A)** Representative images showing H&E stained inguinal white adipose tissue (iWAT) and epididymal white adipose tissue (eWAT), Scale bar 100μm. **(B-C)** Quantification of iWAT **(B)** and epidydimal WAT eWAT **(C)** of HFD mice after drug treatment. (n = 5-6 per group). **(D-F)** Analysis of serum after drug treatment for TG (triglyceride; **D**), TC (total cholesterol; **E**), LDL (low-density lipoprotein; **F**), and HDL (low-density lipoprotein; **G**). (n=6 for all treatment groups), (n=6 for all treatment groups), Data are reported as average ± SEM. One-way ANOVA.

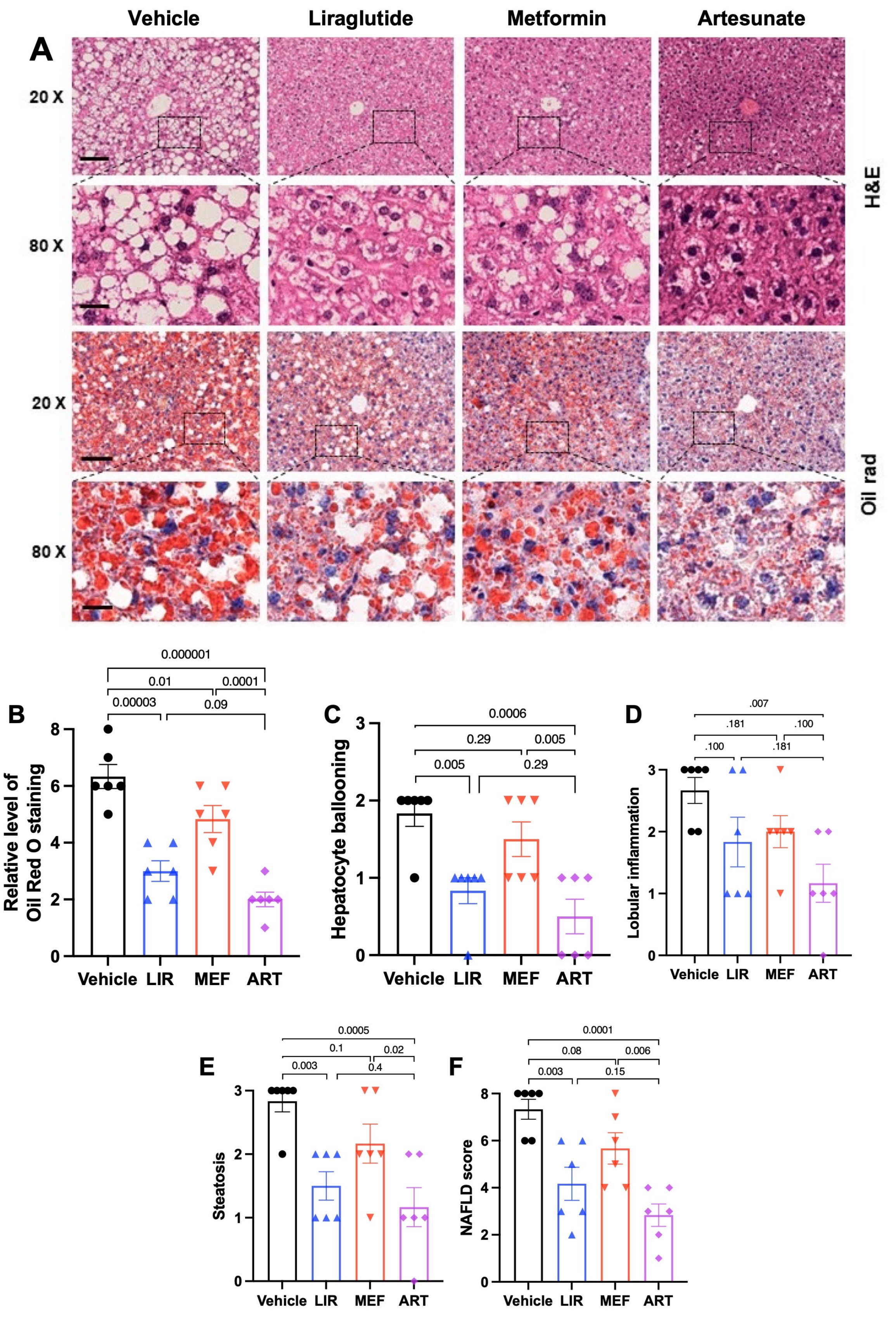

Figure S6 Artesunate (ART) alleviates hepatic steatosis as compared to metformin (MEF) and liraglutide (LIR). **(A)** Histopathological evaluation of the liver from high-fat diet (HFD) mice treated with drugs. Oil red o staining to examine the intracellular lipid accumulation. Scale bar 25µm for 20x magnification & 100µm for 80x magnification. **(B)** Quantification of oil red O positive area. **(C-F)** Histology score for hepatocyte ballooning **(C)**, lobular inflammation **(D),** steatosis **(E),** and non-alcoholic fatty liver disease (NAFLD) activity score **(F)**. (n=6 for all treatment groups), Data are reported as average ± SEM. One-way ANOVA.

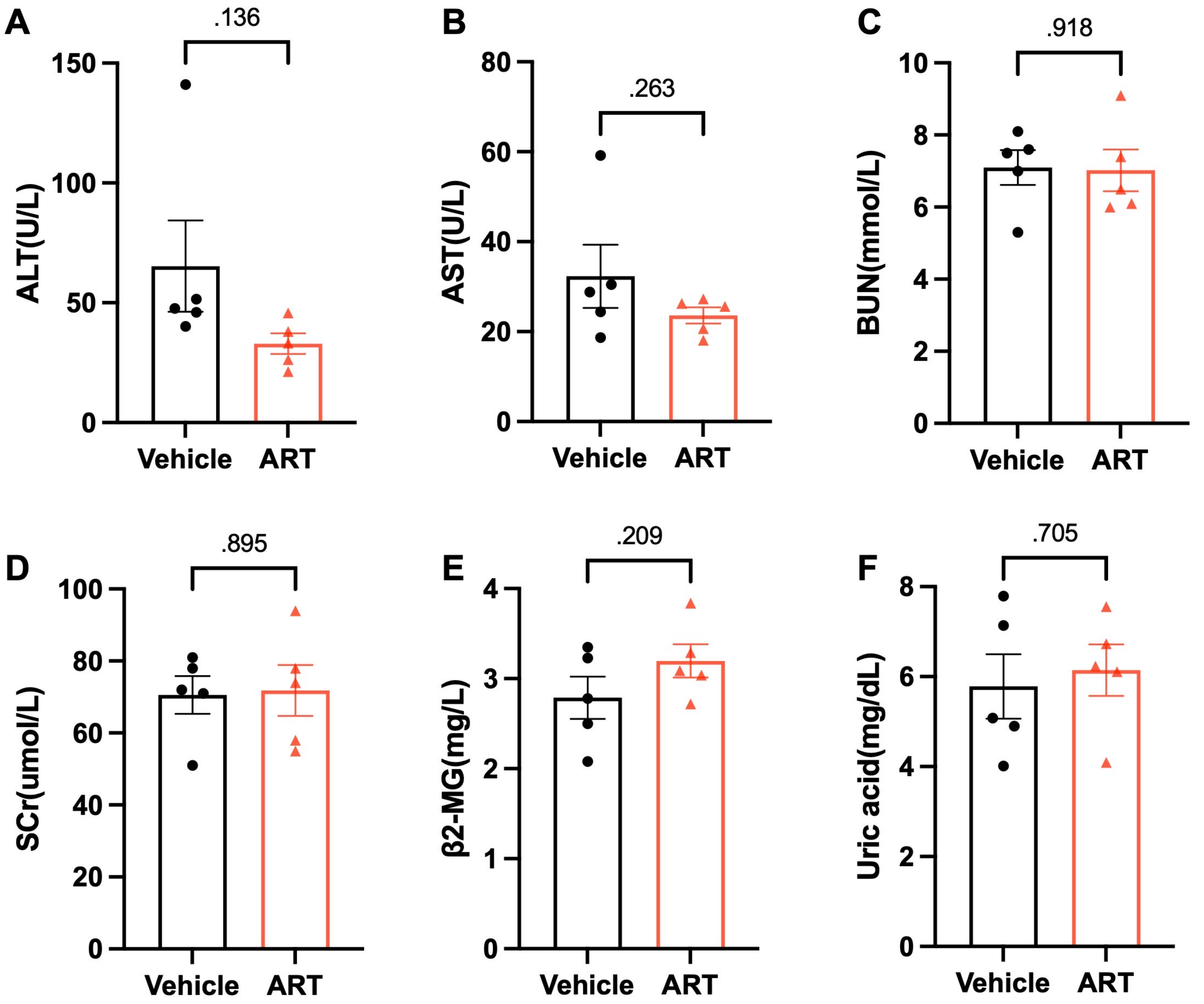

Figure S7. Artesunate (ART) drives weight loss in nonhuman primates without any hepatic damage and renal toxicity. Examination of the plasma for the levels of **(A)** alanine transaminase (ALT), **(B)** aspartate aminotransferase (AST), **(C)** blood urea nitrogen (BUN), **(D)** serum creatinine (SCr), **(E)** beta-2 macroglobulin (β2-MG) and **(F)** uric acid. (n=5 for all treatment groups), Data are reported as average ± SEM. Unpaired *t*-test .

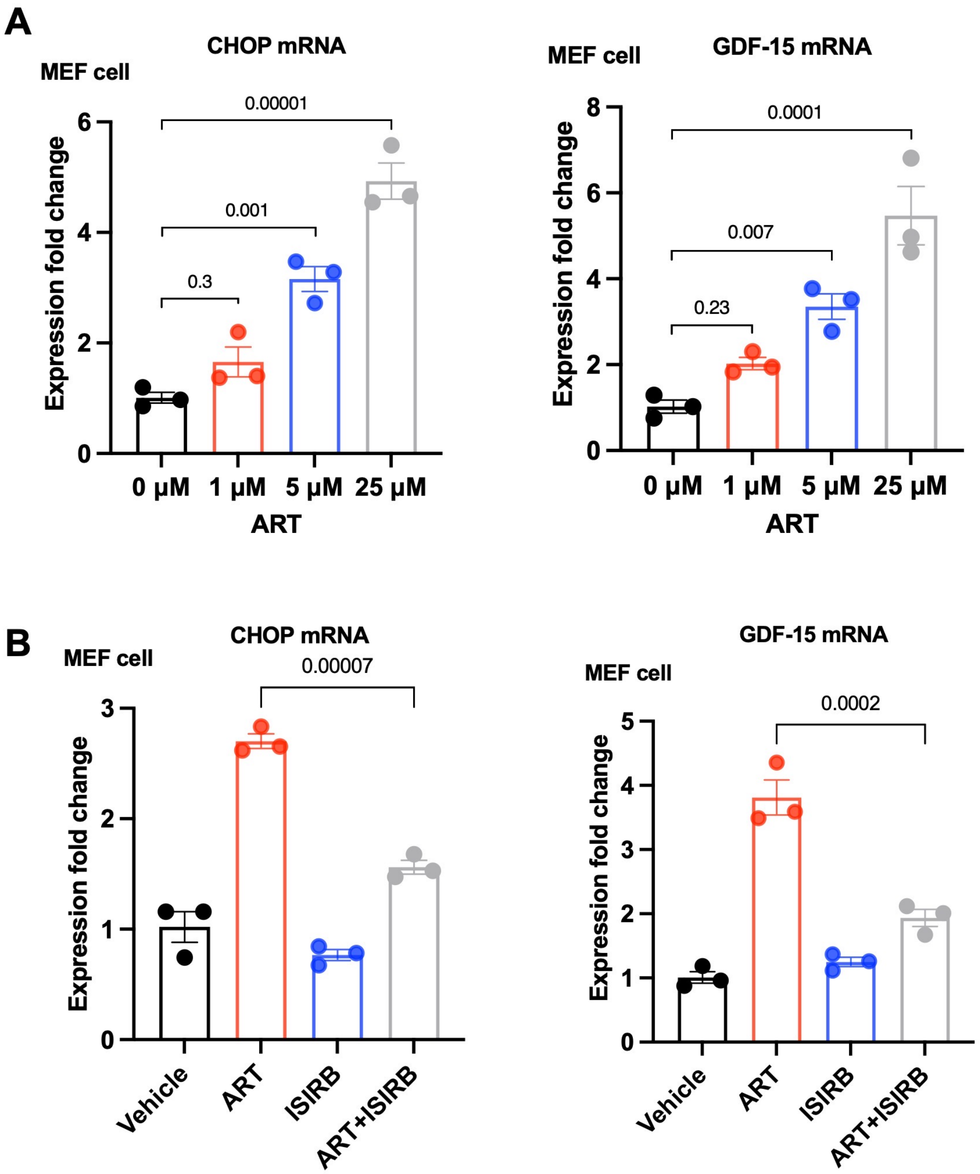

Figure S8. Artesunate (ART) enhances GDF15 expression in mouse embryonic embryonic fibroblasts (MEFs) through integrated stress response (ISR) pathway. qPCR analyses **(A)** of CHOP and GDF15 expression in MEF cells upon 24 h-treatment of ART with different dosages. (n = 3). **(B)** qPCR analyses of CHOP and GDF15 levels in MEF cells cotreated with ART and a small molecule ISIRB which inhibits the activation of ISR. (n=3 for all treatment groups), Data are reported as average ± SEM. One-way ANOVA.

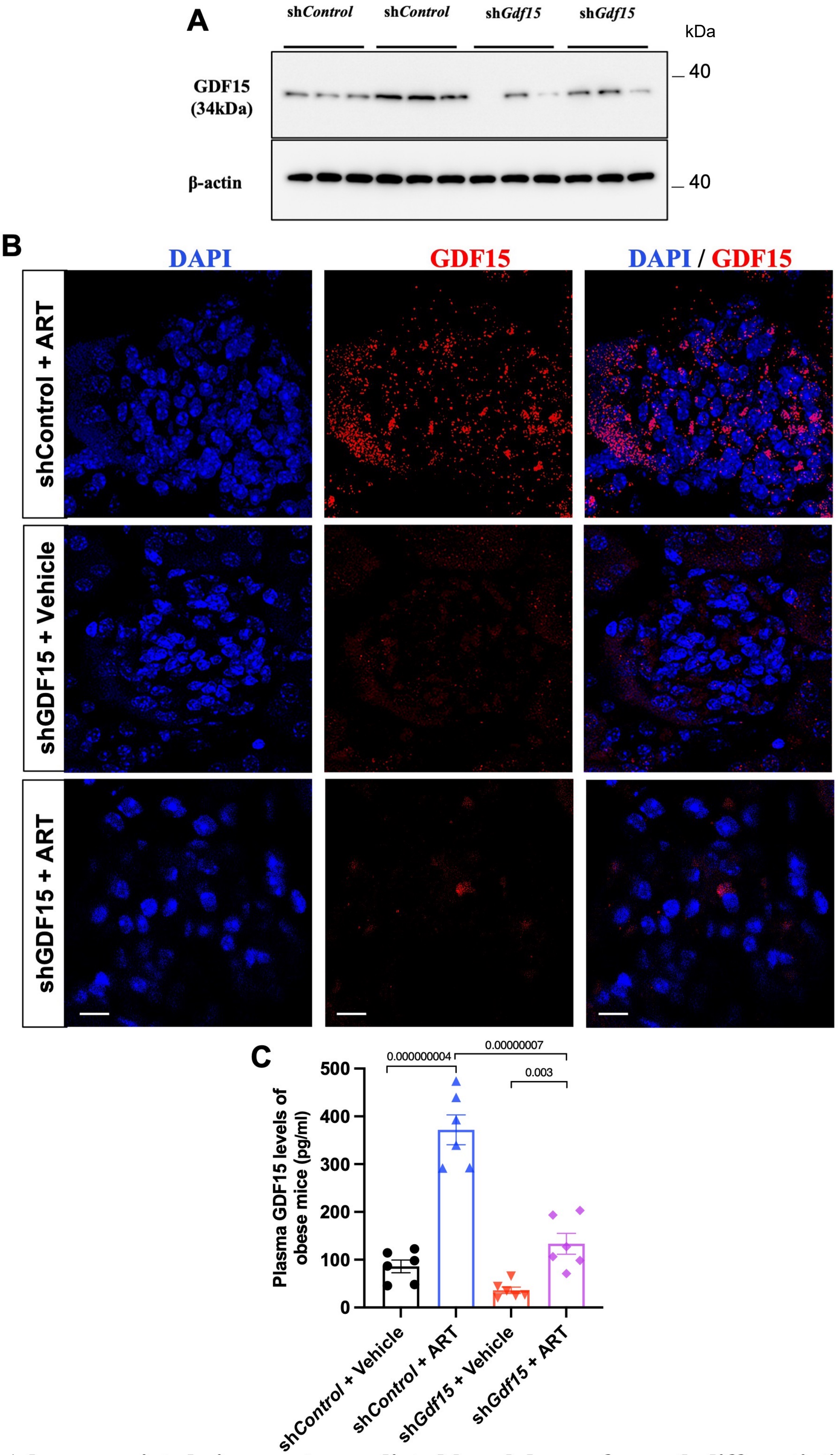

Figure S9. Adeno associated virus vector mediated knockdown of growth differentiation factor 15 (AAV8-shGDF15). **(A)** Western blot analysis from mice hepatic tissue for examining GDF15 expression after AAV8-shGDF15 treatment. **(B)** Immunofluorescent staining of GDF15 (red) in mice kidney tissue after AAV8-shGDF15 treatment. Scale bar 10μm. **(C)** Changes in plasma GDF15 levels after artesunate (ART) treatment in sh*Control* and sh*Gdf15* injected mice. (n=6 for all treatment groups). Data are reported as average ± SEM. One-way ANOVA

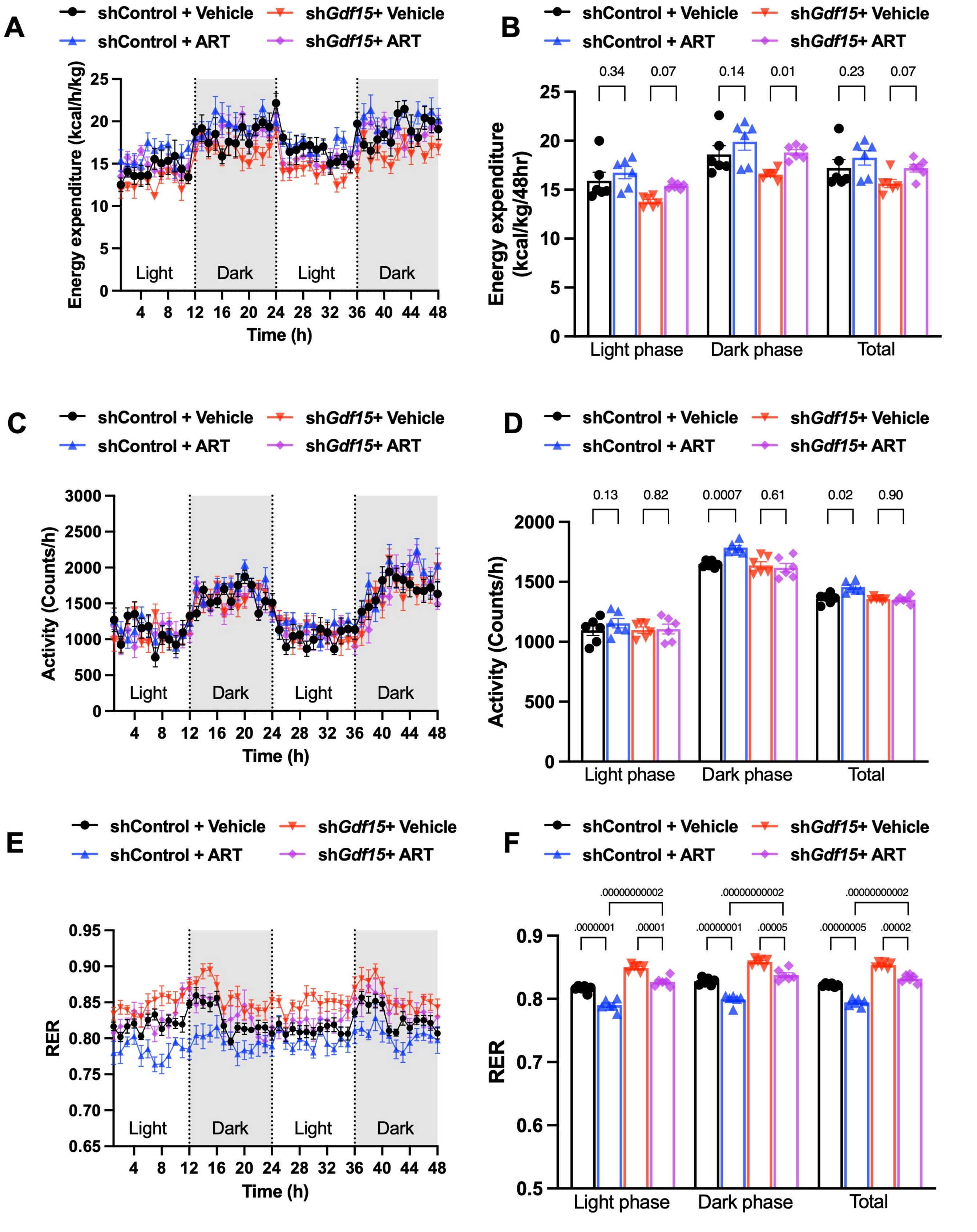

Figure S10. Artesunate (ART) mediated weight loss in diet induced obese mice was independent of energy expenditure changes. (A-F) Energy expenditure changes **(A-B)**, physical activity **(C-D)** and respiratory exchange ratios (RER) quantification **(E-F)** over a 48-hr time-period in vehicle control and ART treated mice with or without *Gdf15* knockdown. (n=6 for all treatment groups), Data are reported as average ± SEM. One-way ANOVA.

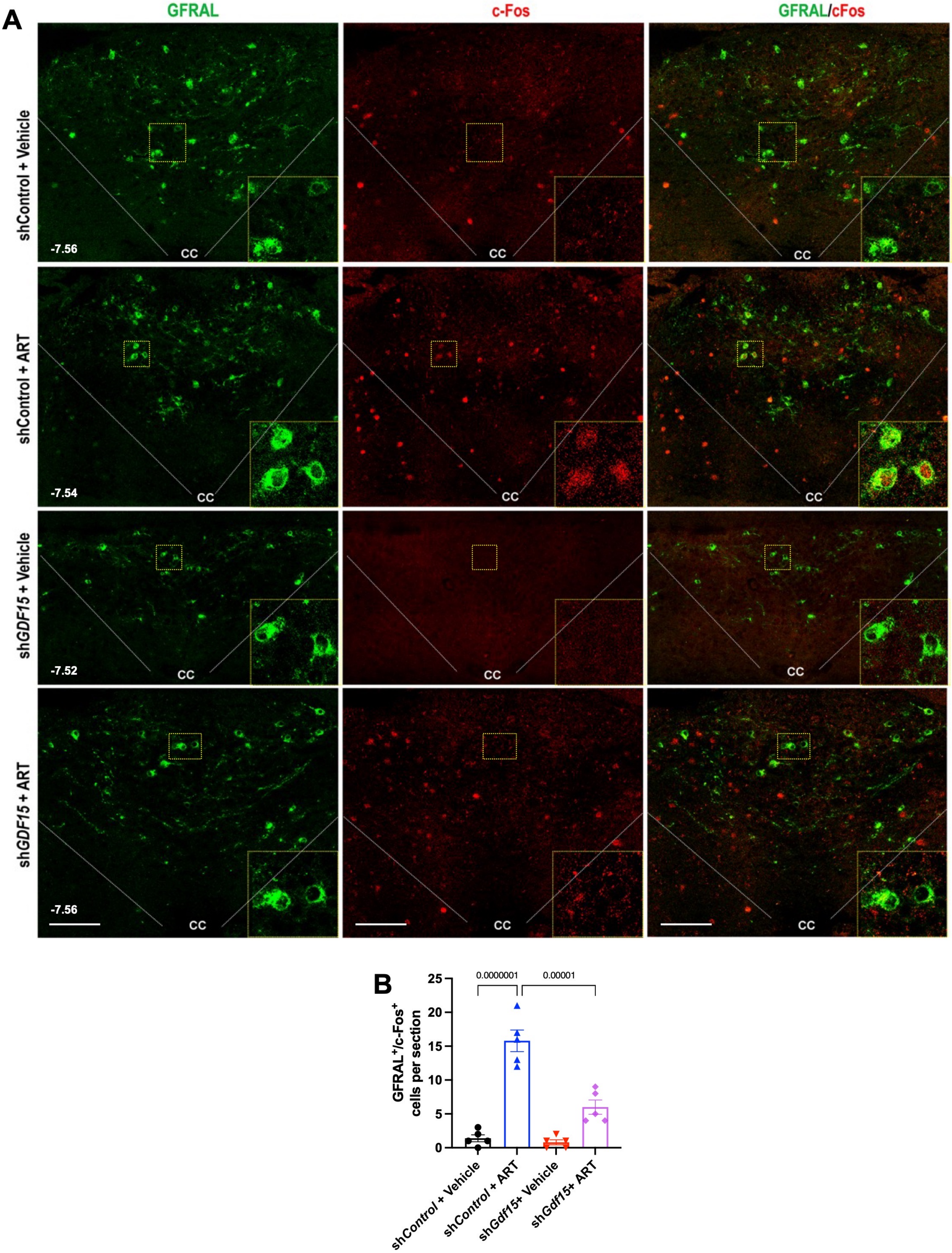

Figure S11. Artesunate (ART) mediated activation of GFRAL neurons is GDF15 dependent. (A) Immunofluorescent staining of GFRAL (green) and c-Fos (red) in area postrema (AP; region of interest indicated by dashed white line) marked above central canal (CC) in the coronal sections within -7.48 to -7.64mm distance from the bregma. Tissue harvest was performed 12 hours after the intraperitoneal administration with ART (20 mg/kg) in shControl and shGDF15 DIO mice. (n= 5 per group). Inset: Magnified view of GFRAL$^+$ neurons (indicated by dashed yellow line). Distance from bregma (in mm) is indicated for each image. Scale bar 100µm. **(B)** Quantification of GFRAL/cFOS double-positive cells in the AP region of tissue samples. Every third section was quantified for each mice/group. (n=5 for all treatment groups), Data are reported as mean ± SEM, one-way ANOVA (B).

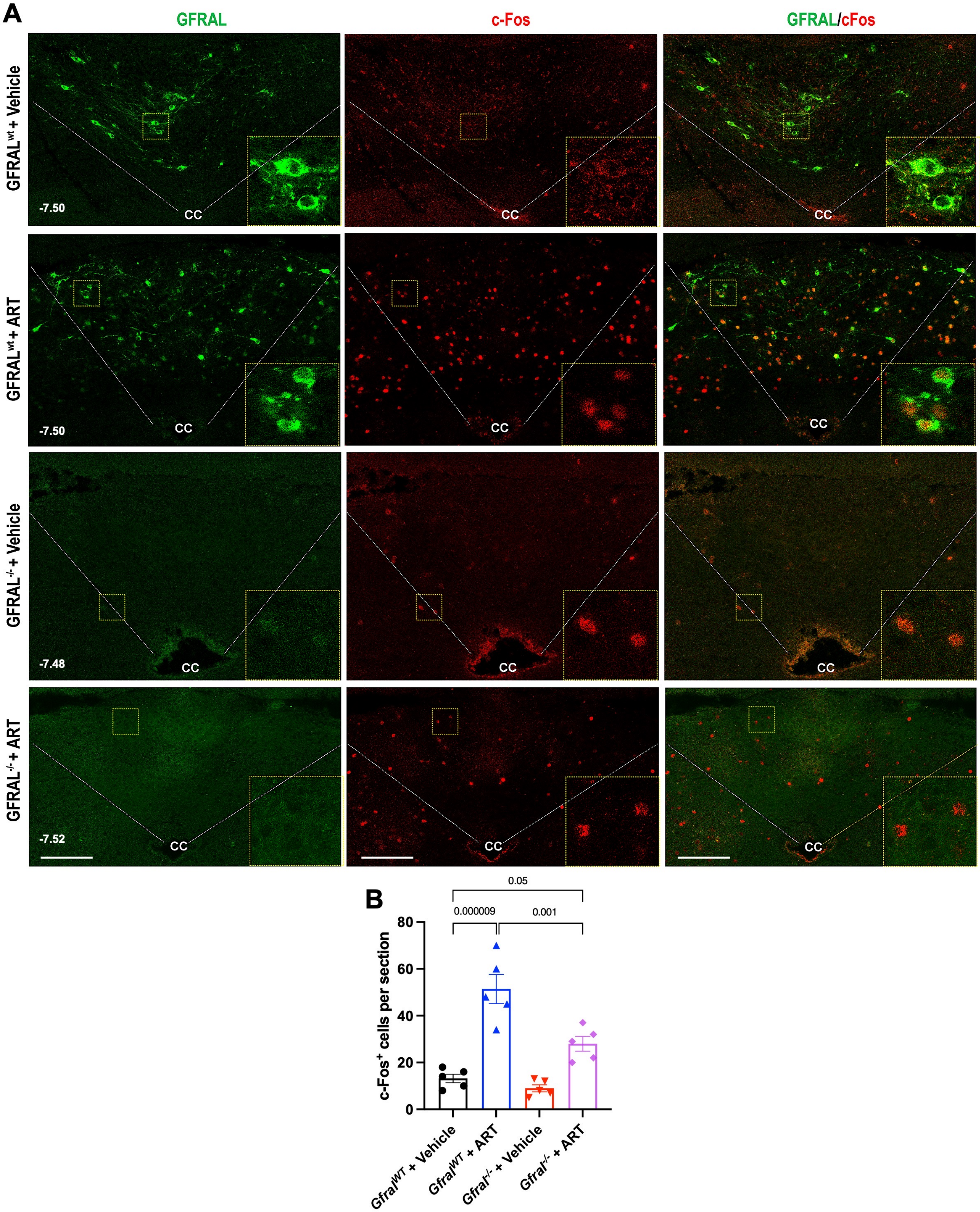

Figure S12. Artesunate (ART) induced neuronal activation is abrogated in Gfral-/- mice. (A) Immunofluorescent staining of GFRAL (green) and c-Fos (red) in area postrema (AP; region of interest indicated by dashed white line) marked above central canal (CC) in the coronal sections within -7.48 to -7.64mm distance from the bregma. Tissue harvest was performed 12 hours after intraperitoneal administration with ART (20 mg/kg) in WT and Gfral-/- mice with DIO. (n= 5 per group). Inset : Magnified view of GFRAL^{+} neurons and cFos^{+} neurons (indicated by dashed yellow line). Distance from bregma (in mm) is indicated for each image. Scale bar 100µm. **(B)** Quantification of cFOS positive cells in the AP region of tissue samples. Every third section was quantified for each mice/group. (n=5 for all treatment groups), Data are reported as mean ± SEM, one-way ANOVA (B).

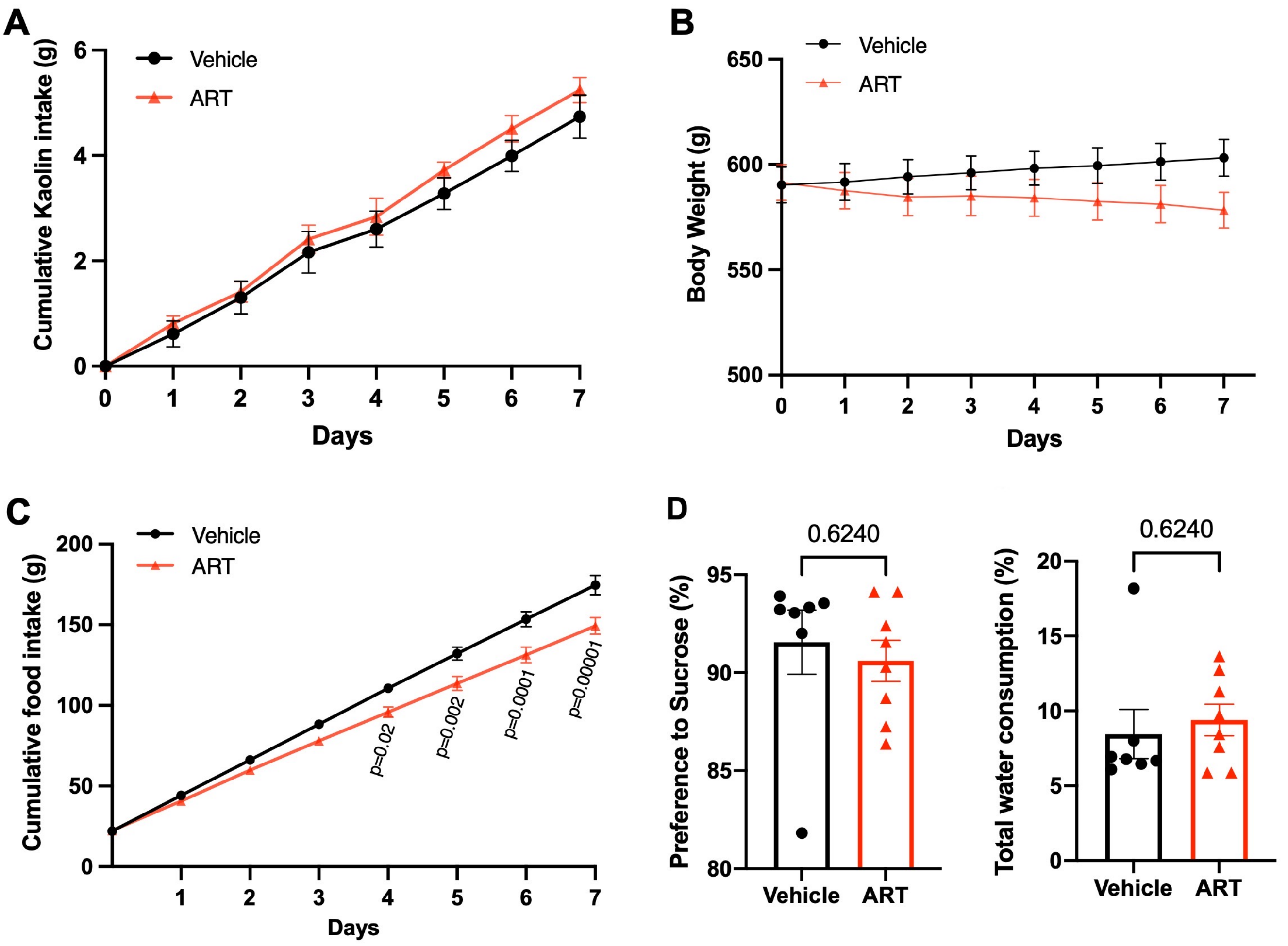

Figure S13. Artesunate (ART) does not induce emesis-like behaviours in rat (A-C) Changes in kaolin intake **(A)**, body weight change **(B)** and cumulative food intake **(C)** of diet induced obesity rats treated with vehicle or ART (n = 5). **(D)** Conditioned taste aversion test showing preference to saccharin after vehicle or ART treatment in mice. (n=7-8 for all treatment groups), Data are reported as average ± SEM. ***P < 0.001; Two-way ANOVA (A-C), unpaired *t*-test (D).

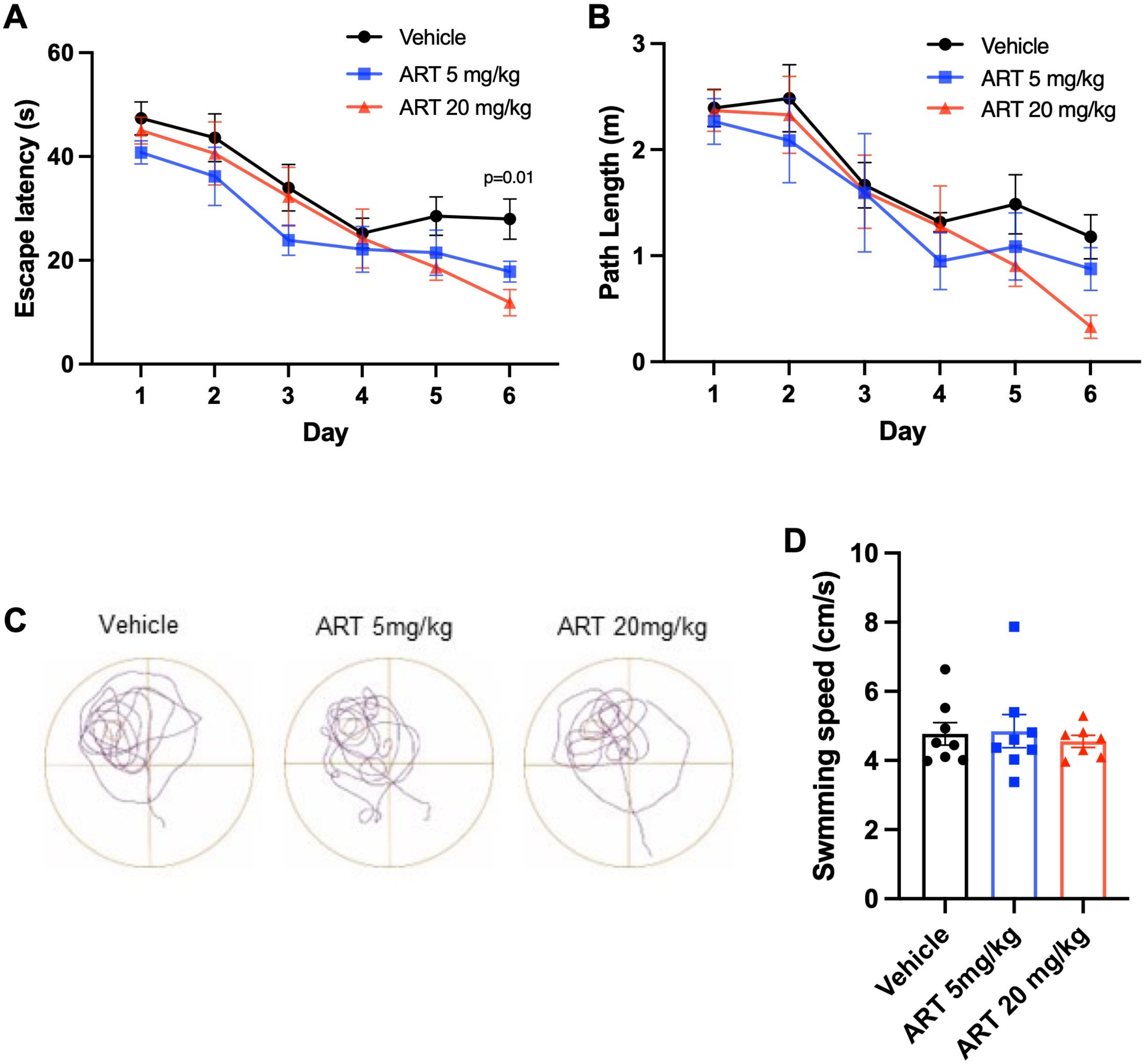

Figure S14. Artesunate (ART) does not induce neurological toxicity in mice. Wild-type mice were daily treated with artesunate (5mg/kg or 20mg/kg) or vehicle over a period of 6 days and their neurobehaviors were assessed by Morris water maze task. **(A-D)** Escape latency **(A)**, total path length **(B)**, representative locomotor trajectories on the day of exploration **(C)** and swimming speed **(D)** of ART-treated mice. (n=8 for all treatment groups), Data are reported as average ± SEM. *P < 0.1. Two-way ANOVA (A-B).

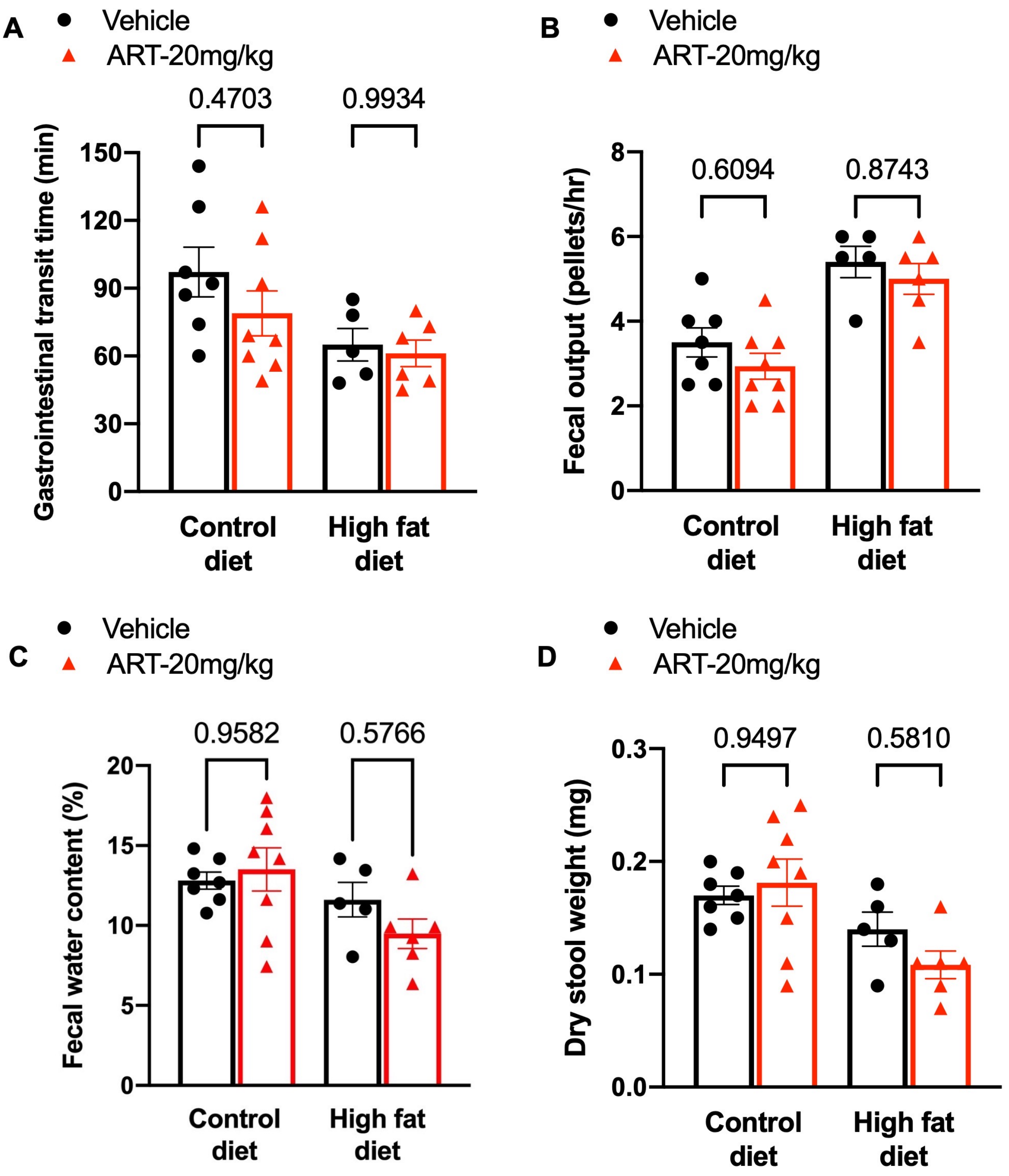

Figure S15. Artesunate (ART) chronic treatment does not induce Gastrointestinal side effects. Gastrointestinal parameters (A-D) Gastrointestinal transit time **(A)**; fecal output calculated as the number of pellets released in 1 hour **(B)**; percentage of fecal water content **(C)**; and dry stool weight **(D)**. (n=5-8 for all treatment groups), Data are reported as average ± SEM. Unpaired *t*-test.

Figure 3D

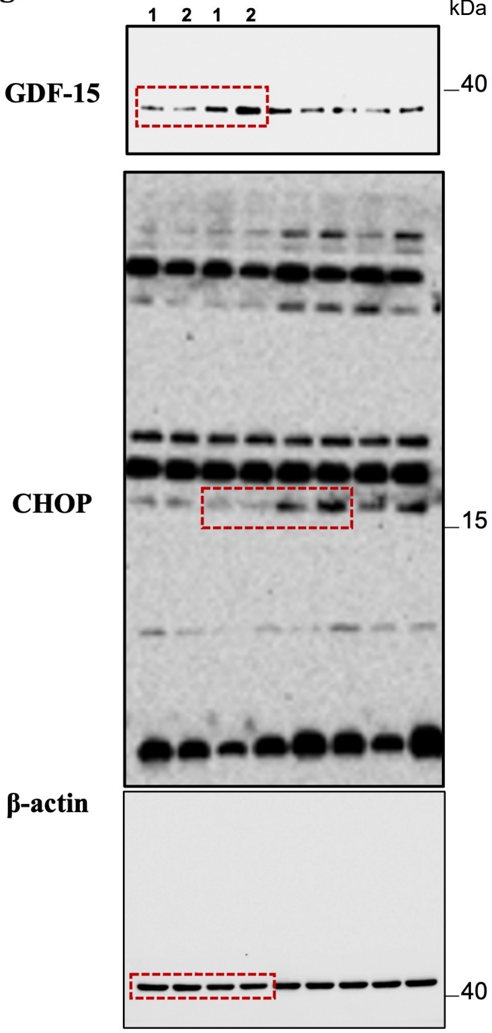

Figure 3E

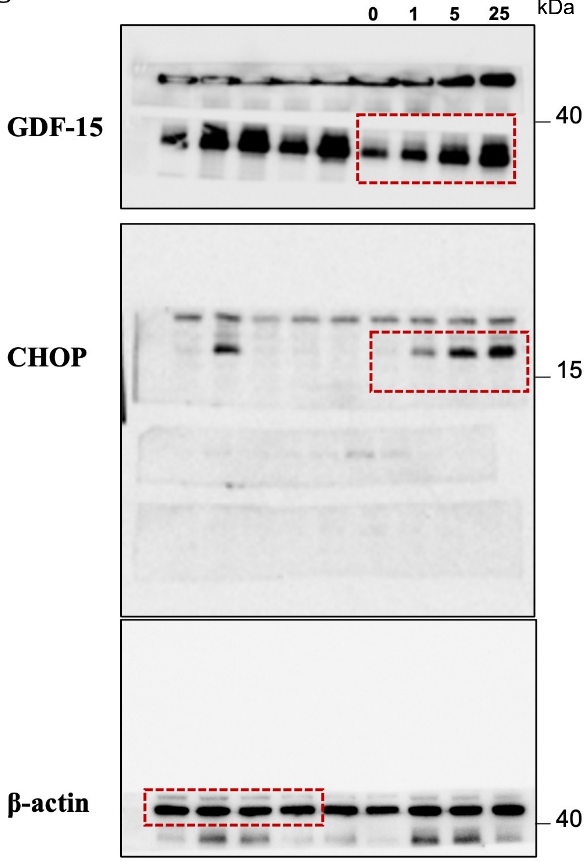

Figure 3H

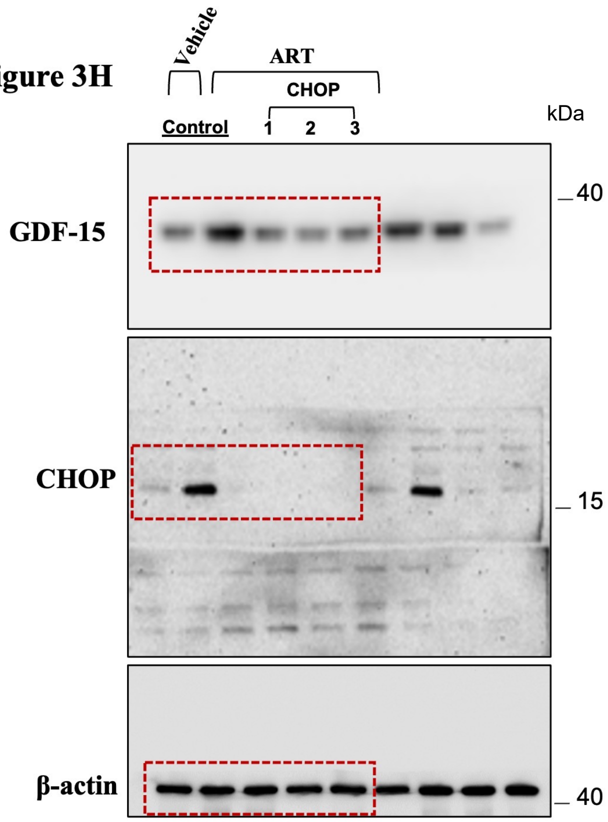

Figure S9A

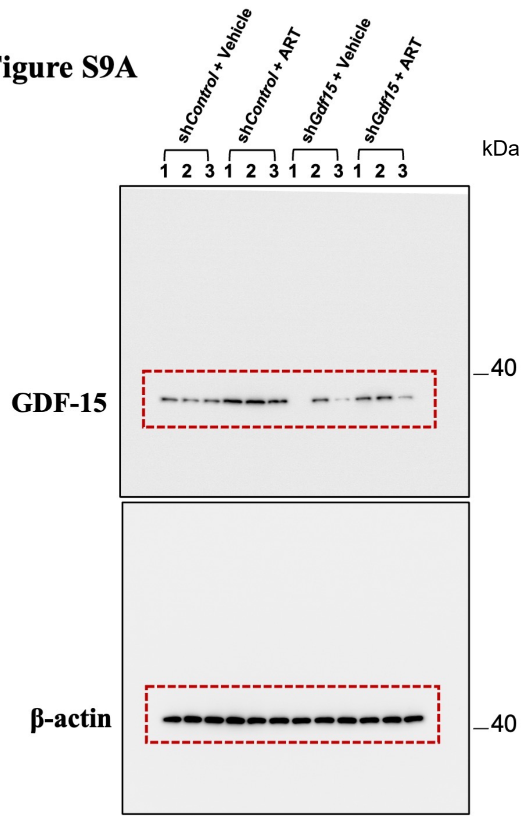

Figure S16. Raw data for western blots. Uncropped images of western blots displayed in figure 3D, 3E, 3H and S9A.

Biochemical Parameters	Control Group	ART-Treated Groups
INS(uIU/ml)	141.76 ± 24.41	80.16 ± 9.05
GLU(mmol/L)	4.94 ± 0.53	3.86 ± 0.28
TG(mmol/L)	1.57 ± 0.28	0.69 ± 0.15
CHOL(mmol/L)	3.12 ± 0.13	2.32 ± 0.22
LDL(mmol/L)	1.78 ± 0.22	0.94 ± 0.12
HDL(mmol/L)	1.46 ± 0.15	0.83 ± 0.09
ALT(U/L)	65.26 ± 19.05	32.92 ± 4.3
AST(U/L)	32.32 ± 7.02	23.6 ± 1.78
GGT(U/L)	52.4 ± 8.52	54.6 ± 5.01
TBIL(umol/L)	2.64 ± 0.36	2.36 ± 0.25
DBIL(umol/L)	0.7 ± 0.15	0.56 ± 0.09
BUN(mmol/L)	7.1 ± 0.48	7.02 ± 0.58
TP(g/L)	73.38 ± 3.16	75.86 ± 2.97
ALB(g/L)	38.58 ± 1.47	30.9 ± 1.06
GLO(g/L)	34.8 ± 2.93	40.28 ± 2.36
A/G	1.14 ± 0.09	1.06 ± 0.1
ALP(U/L)	106 ± 28.31	111.8 ± 33.66
BUN(mmol/L)	7.1 ± 0.48	7.02 ± 0.58
SCr(umol/L)	70.6 ± 5.24	71.8 ± 7.1
β2-MG(mg/L)	2.79 ± 0.23	3.2 ± 0.19
Uric acid(mg/dL)	5.78 ± 0.72	6.14 ± 0.57

Hematological Parameters	Control Group	ART-Treated Groups
RBC(10^12/L)	4.55 ± 0.2	3.41 ± 0.27
HGB(g/dL)	11.06 ± 0.37	11.1 ± 0.57
HCT(%)	36.2 ± 1.21	28.5 ± 2.13
MCV(fL)	79.76 ± 0.92	83.82 ± 1.29
MCH(pg)	24.38 ± 0.53	23.86 ± 0.4
MCHC(g/dL)	30.58 ± 0.34	28.5 ± 0.44
PLT(10^9/L)	566.6 ± 54.77	413 ± 97.61
RDW-SD(fL)	33.2 ± 1	46.1 ± 3.16
RDW-CV(%)	11.54 ± 0.27	16.94 ± 1.17
PDW(fL)	14.36 ± 1.75	12.32 ± 1.03
MPV(fL)	11.8 ± 0.75	10.6 ± 0.44
PCT(%)	0.67 ± 0.07	0.44 ± 0.1
NRBC#(10^9/L)	0.23 ± 0.07	0.33 ± 0.11
NRBC%(%)	0.18 ± 0.07	0.44 ± 0.12
NEUT#(10^9/L)	1.02 ± 0.35	1.55 ± 0.49
LYMPH#(10^9/L)	2.67 ± 0.11	3.05 ± 0.85
MONO#(10^9/L)	1.13 ± 0.2	2.84 ± 0.7
NEUT%(%)	19.68 ± 5.76	28.17 ± 5.85
MONO%(%)	23 ± 2.69	14.92 ± 2.25
EO%(%)	0.7 ± 0.22	0.86 ± 0.17
BASO%(%)	0.2 ± 0.08	0.36 ± 0.02

Table S1 Serum biochemistry and hematology analysis after two weeks of artesunate (ART) treatment in cynomolgus monkey. (n=5 per group), Data are reported as average ± SEM.

	Wild type mice + Control diet		Wild type mice + High fat diet		
**Biochemical Parameters**	**Control Group**	**ART-Treated Groups (20 mg/kg)**	**Control Group**	**ART-Treated Groups (mg/kg)**	
				**Low-Dose (5)**	**High-Dose (20)**
ALT (U/L)	30.00 ± 0.58	27.33 ± 0.88	136.24 ± 5.34	104.2 ± 3.61	90.24 ± 4.07
AST (U/L)	57.83 ± 0.75	58.80 ± 0.51	102.86 ± 1.68	86.74 ± 3.26	71.89 ± 3.64
CK (U/L)	191.33 ± 3.33	136.67 ± 1.86	272.58 ± 7.96	305.33 ± 8.54	331.43 ± 6.89
CR (μmol/L)	14.60 ± 0.36	13.90 ± 0.36	11.58 ± 0.3	10.56 ± 0.35	9.55 ± 0.44
TC (mmol/L)	1.83 ± 0.03	1.93 ± 0.03	5.31 ± 0.15	4.72 ± 0.16	4.17 ± 0.2
TG (mmol/L)	0.90 ± 0.00	0.80 ± 0.00	0.9 ± 0.02	0.79 ± 0.03	0.7 ± 0.03
Urea (mmol/L)	8.67 ± 0.12	7.73 ± 0.03	6.19 ± 0.28	5.89 ± 0.26	6.83 ± 0.29
T-Bil (μmol/L)	0.90 ± 0.12	1.00 ± 0.10	4.49 ± 0.13	4.43 ± 0.13	4.11 ± 0.11
TP (g/L)	51.40 ± 0.35	54.90 ± 0.55	56.11 ± 1.13	53.06 ± 1.12	55.23 ± 1.14
GLU (mmol/L)	11.13 ± 0.03	11.07 ± 0.07	6.5 ± 0.14	5.86 ± 0.16	5.29 ± 0.16
ALB (g/L)	34.67 ± 0.33	37.00 ± 0.58	35.98 ± 0.26	33.93 ± 0.29	32.12 ± 0.33

	Wild type mice + Control diet		Wild type mice + High fat diet		
**Hematological Parameters**	**Control Group**	**ART-Treated Groups (20 mg/kg)**	**Control Group**	**ART-Treated Groups (mg/kg)**	
				**Low-Dose (5)**	**High-Dose (20)**
WBC (10^9/L)	7.98 ± 0.39	9.94 ± 0.50	15.85 ± 0.59	14.49 ± 0.34	12.46 ± 0.45
RBC (10^{12}/L)	6.77 ± 0.34	6.45 ± 0.13	7.94 ± 0.18	7.98 ± 0.19	7.32 ± 0.21
HGB (g/L)	98.00 ± 5.00	92.33 ± 1.76	178.36 ± 2.34	175.15 ± 2.76	165.53 ± 2.67
MCV (fL)	55.53 ± 0.13	56.83 ± 0.27	60.99 ± 0.48	61.95 ± 0.38	60.49 ± 0.41
MCH (pg)	14.50 ± 0.00	14.33 ± 0.03	19.18 ± 0.21	18.56 ± 0.36	18.42 ± 0.32
MCHC (g/L)	261.33 ± 0.33	251.67 ± 2.19	316.56 ± 3.06	317.34 ± 2.22	313.85 ± 3.06
Eosinophil (%)	2.70 ± 0.65	1.80 ± 0.23	1.91 ± 0.42	2.57 ± 0.35	2.21 ± 0.29
Neutrophil%	12.67 ± 1.24	12.50 ± 0.62	10.75 ± 1.57	11.40 ± 0.89	10.82 ± 1.66
Lymphocyte%	81.80 ± 1.00	82.40 ± 0.70	77.20 ± 0.56	78.90 ± 1.52	79.80 ± 1.00
Basophil%	0.23 ± 0.03	0.30 ± 0.00	0.18 ± 0.01	0.22 ± 0.04	0.19 ± 0.03
Monocyte %	1.23 ± 0.20	1.57 ± 0.12	1.44 ± 0.3	1.04 ± 0.15	1.14 ± 0.23
Platelet (10^9/L)	1324 ± 82.52	1331 ± 28.49	1390 ± 75.71	1290 ± 52.00	1324 ± 99.10

Table S2. Serum biochemistry and hematology analysis after two months of artesunate (ART) treatment in mice fed with control diet or high fat diet. (N=3 per group), Data are reported as average ± SEM.
