# Supplementary material for: Artesunate treats obesity in male mice and non-human primates through GDF15/GFRAL signalling axis
Source: Nat Commun. 2024 Feb 3;15:1034. doi: 10.1038/s41467-024-45452-3 (PMC10838268; doi:10.1038/s41467-024-45452-3)
Supplement: Supplementary file 3 — Reporting Summary [file 41467_2024_45452_MOESM3_ESM.pdf]

nature portfolio

Corresponding author(s): Wong Hoi Leong Xavier, Bian Zhaoxiang, Pallavi Asthana

Last updated by author(s): Jan 2, 2024

Reporting Summary

Nature Portfolio wishes to improve the reproducibility of the work that we publish. This form provides structure for consistency and transparency in reporting. For further information on Nature Portfolio policies, see our Editorial Policies and the Editorial Policy Checklist.

Statistics

For all statistical analyses, confirm that the following items are present in the figure legend, table legend, main text, or Methods section.

n/a	Confirmed	
☐	☒	The exact sample size (*n*) for each experimental group/condition, given as a discrete number and unit of measurement
☐	☒	A statement on whether measurements were taken from distinct samples or whether the same sample was measured repeatedly
☐	☒	The statistical test(s) used AND whether they are one- or two-sided<br>*Only common tests should be described solely by name; describe more complex techniques in the Methods section.*
☒	☐	A description of all covariates tested
☐	☒	A description of any assumptions or corrections, such as tests of normality and adjustment for multiple comparisons
☐	☒	A full description of the statistical parameters including central tendency (e.g. means) or other basic estimates (e.g. regression coefficient) AND variation (e.g. standard deviation) or associated estimates of uncertainty (e.g. confidence intervals)
☐	☒	For null hypothesis testing, the test statistic (e.g. *F*, *t*, *r*) with confidence intervals, effect sizes, degrees of freedom and *P* value noted<br>*Give P values as exact values whenever suitable.*
☒	☐	For Bayesian analysis, information on the choice of priors and Markov chain Monte Carlo settings
☒	☐	For hierarchical and complex designs, identification of the appropriate level for tests and full reporting of outcomes
☒	☐	Estimates of effect sizes (e.g. Cohen's *d*, Pearson's *r*), indicating how they were calculated

Our web collection on statistics for biologists contains articles on many of the points above.

Software and code

Policy information about availability of computer code

Data collection	qPCR analyses were performed by the ABI ViiA 7 real-time PCR system (Applied Biosystems).<br>MetaScreen software (v.2.3.15.12) was used to collect data generated from indirect calorimetry.<br>Behavioral data were measured using ANY-maze 6 (Stoeling).
Data analysis	GraphPad Prism V8 for Window OS was used for statistical analyses.<br>qPCR results were analyzed by ViiA 7 Real-time PCR system software (QuantStudio Software v1.6.1)<br>Confocal imaging was performed and analyzed by LAS X microscope software (version 3.7.4_23463)<br>Immunohistochemical, Hematoxylin and eosin staining and Oil-Red O staining analyses were obtained through the PANNORAMIC MIDI II automatic digital slide scanner (3DHISTECH, Budapest, Hungary).<br>Behavioral data were analyses using ANY-maze 6 (Stoeling).

For manuscripts utilizing custom algorithms or software that are central to the research but not yet described in published literature, software must be made available to editors and reviewers. We strongly encourage code deposition in a community repository (e.g. GitHub). See the Nature Portfolio guidelines for submitting code & software for further information.

Data

Policy information about availability of data

All manuscripts must include a data availability statement. This statement should provide the following information, where applicable:

- Accession codes, unique identifiers, or web links for publicly available datasets
- A description of any restrictions on data availability
- For clinical datasets or third party data, please ensure that the statement adheres to our policy

All data supporting the findings of this study are available within the paper and its Supplementary Information. Source data are provided with this paper.

Research involving human participants, their data, or biological material

Policy information about studies with human participants or human data. See also policy information about sex, gender (identity/presentation), and sexual orientation and race, ethnicity and racism.

Reporting on sex and gender	N/A
Reporting on race, ethnicity, or other socially relevant groupings	N/A
Population characteristics	N/A
Recruitment	N/A
Ethics oversight	N/A

Note that full information on the approval of the study protocol must also be provided in the manuscript.

Field-specific reporting

Please select the one below that is the best fit for your research. If you are not sure, read the appropriate sections before making your selection.

- [x] Life sciences
- [ ] Behavioural & social sciences
- [ ] Ecological, evolutionary & environmental sciences

For a reference copy of the document with all sections, see nature.com/documents/nr-reporting-summary-flat.pdf

Life sciences study design

All studies must disclose on these points even when the disclosure is negative.

Sample size	The sample size was estimated with the power of the statistical test performed. We selected the sample size, based on our extensive experience with animal models and endpoints. We make sure that no more animals than necessary were used. Sample size were chosen to generate reproducible results with desirable significance (0.05) and power (>90%).
Data exclusions	No data was excluded from the manuscript.
Replication	All of the experimental results were replicated as indicated in figure legends. For in vitro experiments, each experiment was independently repeated at least three times. Only biological replicates were plotted and used for statistical analyses.
Randomization	Tissues from independently and randomly chosen mice at comparable developmental stages and sexes were collected for analyses and none of the samples was excluded. For the experiments involving transgenic animals, further allocations were based on the genotype of mice. For the experiments with non-human primates, randomly chosen monkeys at comparable developmental stages and sexes (with similar degree of obesity) were collected for analyses and none of the samples was excluded. For in vitro cell experiments, cells of each genotype were parallel seeded and randomly assigned to different treatments.
Blinding	For molecular studies including western blotting and qPCR analyses, the experiments were not blinded, due to careful experimental setup and design. The investigators were not blinded during outcome assessment for the experiments since data acquisition and analysis were done using indicated software.<br>During the phenotyping experiments of transgenic mice and non-human primates, the experiments were performed blinded and the genotype was only disclosed after data analyses. All histology scoring was performed by two blinded researchers. Tissue sections were de-identified and blinded for pathological analysis of histochemical scores. The experimenter was blinded to the experimental groups when doing manual counting of immunohistochemistry, H&E and Oil-Red O images.

Reporting for specific materials, systems and methods

We require information from authors about some types of materials, experimental systems and methods used in many studies. Here, indicate whether each material, system or method listed is relevant to your study. If you are not sure if a list item applies to your research, read the appropriate section before selecting a response.

Materials & experimental systems

n/a	Involved in the study
☐	☒ Antibodies
☐	☒ Eukaryotic cell lines
☒	☐ Palaeontology and archaeology
☐	☒ Animals and other organisms
☒	☐ Clinical data
☒	☐ Dual use research of concern
☒	☐ Plants

Methods

n/a	Involved in the study
☒	☐ ChIP-seq
☒	☐ Flow cytometry
☒	☐ MRI-based neuroimaging

Antibodies

Antibodies used	anti-GFRAL antibody (ab214929, Abcam, 1:100 dilution for immunofluorescent staining); anti-FOS (FOS, 2250, Cell Signaling Technology, 1:200 dilution immunofluorescent staining); anti-GDF15 (27455-1-AP, Proteintech, 1:2,000 dilution for western blot); anti-CHOP (15204-1-AP, Proteintech, 1:2,000 dilution for western blot); anti-β-actin (sc-47778, Santa Cruz, 1:2,000 dilution for western blot); m-IgGκ BP-HRP (sc-516102, Santa Cruz, 1:5,000 dilution); goat anti-rabbit antibody conjugated with HRP (sc-2357, Santa Cruz, 1:5,000 dilution); Alexa Fluor 488-conjugated donkey anti-sheep antibody (A-11015, Invitrogen, 1:500 dilution); Alexa Fluor 594-conjugated goat anti-rabbit (A11012, Invitrogen, 1:500 dilution)
Validation	The validation information of primary antibodies can be found:<br><br>anti-GFRAL antibody: https://www.abcam.com/products/primary-antibodies/gfral-antibody-ab214929.html<br>anti-FOS: https://www.cellsignal.com/products/primary-antibodies/c-fos-9f6-rabbit-mab/2250<br>anti-GDF15: https://www.ptglab.com/products/GDF15-Antibody-27455-1-AP.htm<br>anti-CHOP: https://www.ptglab.com/products/DDIT3-Antibody-15204-1-AP.htm<br>anti-β-actin: https://www.scbt.com/p/beta-actin-antibody-c4<br><br>Secondary antibodies were validated by the manufacturers for the different detection methods. The optimal amounts of the reagents were defined by no minimal to no background signal (Western Blotting, immunofluorescent staining).

Eukaryotic cell lines

Policy information about cell lines and Sex and Gender in Research

Cell line source(s)	Hep-G2 cells were purchased from ATCC (HB-8065). MIHA cell line was a gift from the University of Hong Kong, which was originally purchased from the National Collection of Authenticated Cell Cultures (https://www.cellbank.org.cn/) ; the sex origins of cell lines are unknown.<br>MEF cells were isolated using 13.5 days developed embryos of mice. Cells of both sexes were used for the experiment.
Authentication	Hep-G2 cells from ATCC undergo authentication tests during the accessioning process (STR profiling). This process is described in the online ATCC brochure "Maintaining High Standards in Cell Culture".<br>MIHA cell line was obtained from reliable academic sources (National Infrastructure of Cell Line Resources). No other authentication was performed.
Mycoplasma contamination	The cells were tested negative for mycoplasma contamination
Commonly misidentified lines (See ICLAC register)	No commonly misidentified cell lines were used

Animals and other research organisms

Policy information about studies involving animals; ARRIVE guidelines recommended for reporting animal research, and Sex and Gender in Research

Laboratory animals	C57BL/6J male wild type (WT) mice and Sprague-Dawley rats were from the Laboratory Animal Services Centre of The Chinese University of Hong Kong. Gfral−/− mice on a C57BL/6J background were generated by Cyagen Biosciences Inc. All animals and their borne pups were housed in the animal house at Hong Kong Baptist University and maintained on a 12-h (h) light/dark cycle with constant ambient temperature (22 –24 °C) and humidity (~60%). They were fed with standard laboratory chow, and applied with water ad libitum. All animals were between 6 and 12 weeks old unless otherwise stated in the age group experiments.<br><br>For the non-human primate model of obesity, eight male macaques with a mean age of 12.5 years, MI >40 and body condition scoring >4.5 49were selected. These macaques were housed individually in stainless steel cages and maintained at ambient

	temperature (16-26°C) and humidity (~60%) under a 12-hour (h) light/dark cycle. Breakfast (80 g) and dinner (120 g) were the standard maintenance diet for non-human primates (Jiangsu Synergy Pharmaceutical and Biological Engineering Co., Ltd.), and lunch was apple (70 g). Animals were fed water ad libitum through a waterer.
Wild animals	No wild animals was used.
Reporting on sex	The findings applied to male mice, rats and macaques only. Male subjects are more likely to develop obesity-associated disorders including abnormal liver fat accumulation, non-alcoholic fatty liver disease and liver fibrosis when compared with female subjects before the menopause.
Field-collected samples	This study did not involve samples collected from the fields.
Ethics oversight	All experimental protocols involving mice and rats were reviewed and approved by the Use of Human & Animal Subjects in Teaching & Research (HASC) at Hong Kong Baptist University and in compliance with the Department of Health, Hong Kong.<br><br>For the study involving non-human primates, all husbandry conditions and procedure were approved by Guangzhou Huazhen Bioscience Co., Ltd (IACUC) and conformed to the ethical guidelines of their Institutional Animal Care and Use Committee. The husbandry facilities were accredited by the American Association for Assessment and Accreditation of Laboratory Animal Care (AAALAC).

Note that full information on the approval of the study protocol must also be provided in the manuscript.

Plants

Seed stocks	N/A
Novel plant genotypes	N/A
Authentication	N/A
